# Occupational outcomes after high‐grade or low‐grade brain tumors in childhood: A Swedish, nationwide, registry‐based study

**DOI:** 10.1002/cam4.5464

**Published:** 2022-11-23

**Authors:** Malin Lönnerblad, Eva Berglund, Maria Åberg, Klas Blomgren

**Affiliations:** ^1^ Department of Women's and Children's Health Uppsala University Uppsala Sweden; ^2^ Department of Women's and Children's Health Karolinska Institutet Stockholm Sweden; ^3^ Department of Special Education Stockholm University Stockholm Sweden; ^4^ School of Public Health and Community Medicine, Institute of Medicine, Sahlgrenska Academy University of Gothenburg Gothenburg Sweden; ^5^ Region Västra Götaland Gothenburg Sweden; ^6^ Paediatric Oncology Karolinska University Hospital Stockholm Sweden

**Keywords:** high‐grade tumors, low‐grade tumors, occupational outcomes, pediatric brain tumors, registry‐based study

## Abstract

**Background:**

Survivors of pediatric brain tumors are at high risk of late complications that may affect their daily life in both short‐ and long‐term perspectives.

**Methods:**

In this nationwide registry‐based study we explored the occupational outcomes, including employment, sickness or activity compensation and parental leave, in 452 individuals in Sweden, born 1988–1996, and diagnosed with a brain tumor before their 15th birthday. Their results were compared with 2188 matched controls.

**Results:**

There were significant differences between cases and controls for all assessed variables. The cases had benefitted from sickness or activity compensation 11 times more often than controls (CI 7.90–15.83; *p* < 0.001) between 2005 and 2016. Controls were almost three times more likely to have an employment (OR 0.36; CI 0.28–0.47; *p* < 0.001) and nearly twice as likely to have been on parental leave (OR 0.56; CI 0.39–0.80; *p* = 0.002). Although cases treated for high‐grade tumors typically fared worse than those treated for low‐grade tumors, significant differences for all assessed variables were also observed for cases treated for a low‐grade tumor compared with controls.

**Conclusions:**

Our findings emphasize the need for follow‐up programs for all brain tumor diagnoses, not only those known to be at most risk. This is evident, for example, from the high number of cases who received sickness or activity compensation.

## Graphical abstract

Our findings showed that between the years 2011‐2016 the proportion of individuals treated for brain tumors (pediatric brain tumor survivors, PBTS) born 1988‐1996 were employed to a significantly lower extent compared with matched controls born the same years. They received sickness or activity compensation at least ten times more often than controls and around twice as many controls were on parental leave compared with individuals treated for brain tumors.

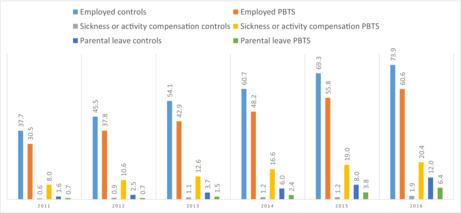

## 1 INTRODUCTION

During the period 1984–2005, the brain tumor incidence for children in Sweden below the age of 15 was 4.4 out of 100,000 and the overall survival rate was close to 80%.^1^ It is well known that children treated for brain tumors, hereafter referred to as pediatric brain tumor survivors (PBTS), are at high risk of late complications.^2^, ^3^, ^4^ Neurocognitive impairments, for example reduced processing speed, working memory and attention,^3^, ^5^, ^6^, ^7^ are common and often combined with mental fatigue.^8^, ^9^, ^10^, ^11^ Together with cardiovascular and respiratory complications,^12^, ^13^ these impairments affect their daily lives, including occupational outcomes. There is an association between young age at diagnosis and more severe late complications,^3^, ^14^, ^15^ and the resulting problems may increase over time.^3^, ^7^, ^16^ Several studies have found that females may be more severely affected than males,^3^, ^17^, ^18^, ^19^ although other studies have shown the opposite,^20^, ^21^ or no difference at all.^22^, ^23^ These differing results may be due to factors such as age at diagnosis, tumor size, and the type of skills examined.^21^, ^24^ PBTS treated for high‐grade tumors are at particular risk of late complications,^25^, ^26^ and there is accumulating evidence that PBTS treated for low‐grade tumors are also at risk.^27^, ^28^, ^29^, ^30^ De Boer and colleagues found in a meta‐analysis that PBTS are at five times higher risk for unemployment.^31^ Other studies have shown that PBTS tend to live with their parents longer, were more often single, and got married less often than adults who had not experienced childhood brain tumors.^32^, ^33^ However, there is great inter‐individual variation, such that some individuals do not experience difficulties at all, while others need considerable extra help in their daily lives.^34^

Since cancer treatments have changed and treatment results has improved over the years,^35^, ^36^ it is important to update our understanding of occupational and other outcomes accordingly. The aim of the present study was to explore occupational outcomes in Swedish teenagers and young adults born in 1988–1996 and treated for a brain tumor during childhood. We also wanted to explore possible differences due to sex, age at diagnosis, parents' education, and tumor type. Nearly 40% of all pediatric brain tumors are low‐grade astrocytomas, typically treated with surgery and/or chemotherapy, which in contrast to radiation therapy are treatments less prone to cause late complications. Optic pathway gliomas are typically low‐grade, and treated with chemotherapy only, but due to their effects on vision, they may affect academic performance and occupational outcomes for reasons unrelated to the treatment. Craniopharyngiomas are also low‐grade tumors, but their treatments can be difficult, and significant morbidities are associated with both the tumor and treatments, including partial loss of the visual fields and profound endocrine effects because of its proximity to the pituitary gland. Hence, even though the treatment modalities of low‐grade tumors are considered to carry a lesser risk of late complications, very few studies have investigated the occupational outcomes for these subgroups.

## 2 MATERIALS AND METHODS

In this study, we have explored the occupational outcomes of 452 individuals in Sweden born 1988–1996 and who were diagnosed with a brain tumor before their 15th birthday, including individuals with relapses. Information about tumor type and medical background factors was obtained from the Swedish Childhood Cancer Registry (Table 1). Information about radiation therapy is unfortunately often incomplete or missing in the registry. For example, available information is often in a free text format, the dose given is not always included, and it is not always clear whether radiation therapy was craniospinal or focal. Children under the age of 4–5 years with high‐grade tumors, such as medulloblastoma or AT/RT, are typically excluded from cranial radiation therapy and may be treated with intrathecal chemotherapy instead, but this has also been shown to affect neurocognitive outcomes.^23^, ^37^ For these reasons, we decided to use diagnosis as a surrogate marker for treatment intensity, assuming that grade III to IV tumors receive radiotherapy or intrathecal chemotherapy to a much greater extent than grade I and II tumors.

**TABLE 1:** Medical characteristics of included pediatric brain tumor survivors (PBTS)

Tumor classification	PBTS *N*	High‐grade *N*	Low‐grade *N*
Ependymomas	27	9	18
Choroid plexus tumors	10	1	9
Astrocytomas	163	7	156
Optic pathway gliomas	40	—	40
Embryonal tumors (e.g., medulloblastoma and PNET^a^)	52	52	—
Oligodendrogliomas	11	1	10
Mixed and unspecified gliomas	11	3	8
Neuroepithelial glial tumors of uncertain origins	4	—	4
Pituitary adenomas and carcinomas	10	2	8
Tumors of the sellar region (craniopharyngioma)	31	—	31
Pineal parenchymal tumors	8	3	5
Neuronal and mixed neuronal‐glial tumors	38	1	37
Meningiomas	11	—	11
Specified intracranial/intraspinal tumors	1	—	1
Unspecified intracranial/intraspinal tumors	8	—	8
Other specified/unspecified tumors	2	—	2
Nerve sheath tumors	5	—	5
Germ cell tumors	8	1	7
Non‐CNS^b^ tumors by definition	12	—	12

*Note*: Tumor classification according to the Swedish Childhood Cancer Registry. High‐grade tumors, WHO III‐IV (*n* = 80) and low‐grade tumors, WHO I‐II (*n* = 372).

^a^ Primitive neuro‐ectodermal tumors.

^b^ Central nervous system.

Coded data linked to each individual's unique personal identification number were sent from the Swedish Childhood Cancer Registry to Statistics Sweden and matched to about five unique controls per case by birth year, sex, and place of living at diagnosis, yielding 2188 controls. Children diagnosed with any cancer during the study years were not eligible as controls. The place of living was listed on the parish level.^38^ From Statistics Sweden, we obtained data about occupational outcomes, whether Swedish was their first or second language at school, and information about parents' education (Table 2). As several studies have shown a positive correlation between parents' education and the students' academic achievements,^39^, ^40^, ^41^, ^42^ it is important to be able to control for this.

**TABLE 2:** Included participants

	PBTS *N* (%)	High‐grade *N*	Low‐grade *N*	Controls *N* (%)	Chi‐squared tests, *p*‐value
All	452	80	372	2188	*p* = 0.976
Females	218 (48.2)	37	181	1057 (48.3)	
Males	234 (51.8)	43	191	1131 (51.7)	
Age at diagnosis					
Females 0–5 years	80 (36.7)	20	60		
Females 6–9 years	48 (22.0)	10	28		
Females 10–14 years	90 (41.3)	7	83		
Age at diagnosis					
Males 0–5 years	83 (35.5)	14	69		
Males 6–9 years	63 (26.9)	8	55		
Males 10–14 years	88 (37.6)	21	67		
Swedish as the first or second language					*p* = 0.653
As the first language	429 (94.9)			2092 (95.6)	
As the second language	23 (5.1)			96 (4.4)	
Mothers' education					*p* = 0.345
Low (school years 1–9 or less)	36 (8.0)			218 (10.0)	
Medium (upper secondary school)^a^	221 (48.9)			1083 (49.5)	
High (higher education)	193 (42.7)			881 (40.3)	
No information about education	2 (0.4)			6 (0.3)	
Fathers' education					*p =* 0.292
Low (school years 1–9 or less)	77 (17.0)			349 (16.0)	
Medium (upper secondary school)^a^	218 (48.2)			1151 (52.6)	
High (higher education)	148 (32.7)			660 (30.2)	
No information about education	9 (2.0)			28 (1.3)	

*Note*: Pediatric brain tumor survivors (PBTS; *n* = 452) with high‐ or low‐grade tumors and controls (*n* = 2188). Chi‐squared tests for differences between the cases and controls.

^a^ Until 1994, upper secondary school could be two (mainly vocational educations) or three (theoretical educations) years in Sweden.

All data handled were deidentified and only Statistics Sweden has the key code. The same cohort has been investigated in three previous articles comparing final grades from the last year of compulsory school in theoretical^28^ and practical and esthetic^29^ school subjects, and the national test grades that preceded the final grades in the three theoretical subjects^43^ between PBTS and controls. Only children with grades from the regular school system were included in these studies since the compulsory schools for children with intellectual disabilities did not have a uniform grading system during the study time period and were therefore excluded. During the years 2003–2012, the number of children in Sweden annually attending compulsory schools for children with intellectual disabilities ranged between 1.4–1.5%,^44^ and one Swedish study estimated that only 3% of PBTS were enrolled in such schools.^27^

In the present study we have also excluded PBTS and controls that were no longer alive in 2016 (PBTS, *n* = 23; 4.84%; Controls *n* = 9; 0.4%). For more information about inclusion and exclusion, see Lönnerblad et al., 2021.^43^ All the parents of the included PBTS have given written permission for the child to be included in the Swedish Childhood Cancer Registry, and all PBTS were asked again when they turned 18 if they wanted to remain in the registry. Ethical approval for this study was given by the Regional Ethical Review Board in Stockholm (no 2017/995–31/5).

Occupational outcomes include information about employment, sickness or activity compensation, and parental leave from the years 2005–2016. The year 2005 was the first year with available data from this registry for individuals born in 1988. In 2005, the participants were 9–17 years old, and in 2016 they were 20–28 years old. *Employment* indicates whether a person, from the age of 16, has been employed or not (including self‐employment). From age 19, some individuals were eligible for *sickness or activity compensation*.^45^ Decisions on sickness or activity compensation are based on medical reports, and this support is aimed for individuals who because of sickness or disability are unable to work or to work full‐time or who need to prolong their basic education. *Parental leave* allows parents in Sweden, both females and males, to receive an income from the state when caring for their children instead of working, studying, or looking for a job. Since 2002, parents have in total 480 days per child to share, whereof 60 days are reserved for each parent, such that if one parent uses less than 60 days, a maximum of 420 days remain for the other parent.^46^

## 3 STATISTICAL METHODS

IBM SPSS Statistics for Windows, Version 28.0.1.0 (New York, USA) was used for statistical analyses. *P*‐values below 5% were considered statistically significant. The differences between PBTS and controls, between females and males, and any possible interaction effect between these are described with odds ratios (OR) using 95% confidence intervals and tested with logistic regressions. The variables PBTS/control and female/male were used as independent variables, and employment, sickness or activity compensation, and parental leave were used as dependent variables. Possible interaction effects between PBTS or controls and mothers' and fathers' education were also tested. Thereafter, we performed logistic regressions with only PBTS included. In these logistic regressions, tumor grade [high‐grades tumors, WHO III‐IV, versus low‐grade tumors, WHO I‐II, and age at diagnosis (age groups 0–5, 6–9, and 10–14 years)] were used as independent variables, while employment, sickness or activity compensation, and parental leave, were used as dependent variables. Differences between the four subgroups of low‐grade tumors (low‐grade astrocytomas, optic pathway gliomas, craniopharyngiomas, and neuronal and mixed neuronal‐glial tumors) were compared with controls using Pearson's Chi‐square test and described as odds ratios (OR) and 95% confidence intervals (CI). These were the subgroups with enough subjects to enable statistical analysis. To ensure participant integrity, numbers equal to or below five in Table 5 are only marked with <5. OR were not calculated in those cases due to low case numbers. We also compared the PBTS and control groups year by year for employment, sickness or activity compensation, and parental leave using Chi‐square tests.

## 4 RESULTS

### 4.1 Employment

The employment rates between 2005 and 2016 showed that PBTS were less frequently employed compared with controls (OR 0.36, *p* < 0.001; Table 3). There was a significant difference for sex, with fewer males than females having been employed, but there was no interaction effect between sex and being PBTS or control (*p* = 0.183), i.e., sex had the same impact on employment for both PBTS and controls. Both mothers' and fathers' education affected the results, but there was no interaction effect between parents' education and being employed, neither for PBTS, nor for controls (mothers' education *p* = 0.922; fathers' education *p* = 0.213). Thus, parents' education had the same impact for both PBTS and controls. There were no significant differences between those treated at different ages. PBTS treated for a high‐grade tumor were employed to a lesser extent than those treated for a low‐grade tumor. Still, PBTS with low‐grade astrocytomas, optic pathway gliomas, craniopharyngiomas, or neuronal and mixed neuronal‐glial tumors were employed at a lower rate compared with controls.

**TABLE 3:** Employment any time between 2005–2016

Employment	*N* (%)	OR (95% CI; *p*)
PBTS vs. all controls	334 (73.9) vs. 1937 (88.5)	0.36 (0.28–0.47; *p* < 0.001)
Sex (all boys vs. all girls)	1142 (83.7) vs. 1129 (88.5)	0.66 (0.52–0.82; *p* < 0.001)
Female PBTS vs. female controls	165 (75.7) vs. 964 (91.2)	0.30 (0.21–0.44; *p* < 0.001)
Male PBTS vs. male controls	169 (72.2) vs. 973 (86.0)	0.42 (0.30–0.59; *p* < 0.001)
Mothers' education		*p* < 0.001
Fathers' education		*p* < 0.001
Differences between PBTS age groups		*p* = 0.843
High‐grade tumors vs. low‐grade tumors	46 (57.5) vs. 288 (77.4)	0.40 (0.24–0.66; *p* < 0.001)
High‐grade tumors. vs. all controls	46 (57.5) vs. 1937 (88.5)	0.18 (0.11–0.28; *p* < 0.001)
Low‐grade tumors vs. all controls	288 (77.4) vs. 1937 (88.5)	0.44 (0.34–0.59; *p* < 0.001)
Low‐grade astrocytomas vs. all controls	127 (81.4) vs. 1937 (88.5)	0.57 (0.37–0.87; *p* = 0.008)
Optic pathway gliomas vs. all controls	28 (70.0) vs. 1937 (88.5)	0.30 (0.15–0.60; *p* < 0.001)
Craniopharyngiomas vs. all controls	20 (64.5) vs. 1937 (88.5)	0.24 (0.11–0.50; *p* < 0.001)
Neuronal and mixed neuronal‐glial tumors vs. all controls	29 (76.3) vs. 1937 (88.5)	0.42 (0.20–0.89; *p* = 0.020)

*Note*: Number (*N*), percentage, odds ratio (OR), 95% confidence interval (CI), and significance (*p*) for differences between pediatric brain tumor survivors (PBTS) and controls.

### 4.2 Sickness or activity compensation

PBTS were more than 11 times more likely to obtain sickness or activity compensation than controls (Table 4). There were no significant differences between females and males, and no interaction effect between sex and being PBTS or control (*p* = 0.128). Neither was there a significant effect of parents' education, nor was there an interaction effect between parents' education and receiving sickness or activity compensation for PBTS and controls (mothers' education *p* = 0.743; fathers' education *p* = 0.832), meaning that this was similar for both groups.

**TABLE 4:** Sickness or activity compensation any time between 2005–2016

Sickness or activity compensation	*N* (%)	OR (95% CI; *p*)
PBTS vs. all controls	101 (22.5) vs. 55 (2.5)	11.18 (7.90–15.83; *p* < 0.001)
Sex (all boys vs. all girls)	74 (5.4) vs. 82 (6.4)	0.82 (0.58–1.15; *p* = 0.243)
Female PBTS vs. female controls	57 (26.1) vs. 25 (2.4)	14.62 (8.88–24.06; *p* < 0.001)
Male PBTS vs. male controls	44 (18.8) vs. 30 (2.2)	8.50 (5.21–13.86; *p* < 0.001)
Mothers' education		*p* = 0.888
Fathers' education		*p* = 0.646
Differences between PBTS age groups		*p* = 0.130
High‐grade tumors vs. low‐grade tumors	31 (38.8) vs. 70 (18.8)	2.87 (1.70–4.86; *p* < 0.001)
High‐grade tumors. vs. all controls	31 (38.8) vs. 55 (2.5)	24.54 (14.54–41.41; *p* < 0.001)
Low‐grade tumors vs. all controls	70 (18.8) vs. 55 (2.5)	8.99 (6.19–13.06; *p* < 0.001)
Low‐grade astrocytomas vs. all controls	25 (16.0) vs. 55 (2.5)	7.40 (4.47–12.26; *p* < 0.001)
Optic pathway gliomas vs. all controls	9 (22.5) vs. 55 (2.5)	11.26 (5.12–24.78; *p* < 0.001)
Craniopharyngiomas vs. all controls	12 (38.7) vs. 55 (2.5)	24.49 (11.33–52.94; *p* < 0.001)
Neuronal and mixed neuronal‐glial tumors vs. all controls	6 (15.8) vs. 55 (2.5)	7.27 (2.92–18.10; *p* < 0.001)

*Note*: Number (*N*), percentage, odds ratio (OR), 95% confidence interval (CI), and significance (*p*) for differences between pediatric brain tumor survivors (PBTS) and controls.

PBTS treated for a high‐grade tumor received sickness or activity compensation almost three times more frequently compared with those treated for a low‐grade tumor (Table 4). There were also significant differences between PBTS treated for low‐grade tumors compared with the control group. PBTS treated for craniopharyngiomas, a low‐grade tumor, displayed the same high rate of sickness or activity compensation as PBTS treated for high‐grade tumors (OR 24.49 vs. 24.54; Table 4). PBTS treated for optic pathway gliomas received sickness or activity compensation more often than the average for PBTS treated for low‐grade tumors (OR 11.26 vs. 8.99; Table 4).

### 4.3 Parental leave

Significantly fewer PBTS had been on parental leave compared with controls (OR 0.56; *p* = 0.002; Table 5). There were significantly fewer males than females who had been on parental leave, but this was the same for both PBTS and controls, as there was no interaction effect (*p* = 0.332). Mothers' and fathers' education affected the results significantly and equally for both PBTS and controls, as there were no interaction effects (mothers' education *p* = 0.287; fathers' education *p* = 0.268). Neither were there any significant differences within the group of PBTS treated at different ages, nor between PBTS treated for a high‐grade or a low‐grade tumor. PBTS treated for low‐grade tumors had significantly less often been on parental leave compared with controls, but those treated for low‐grade astrocytomas did not differ significantly in this respect compared with controls. The numbers of PBTS being on parental leave and treated for high‐grade tumors, optic pathway gliomas, craniopharyngiomas, or neuronal and mixed neuronal‐glial tumors were five or fewer, and therefore ORs were not calculated.

**TABLE 5:** Parental leave any time between 2005–2016

Parental leave	*N* (%)	OR (95% CI; *p*)
PBTS vs. all controls	36 (8.0) vs. 293 (13.4)	0.56 (0.39–0.80; *p =* 0.002)
Sex (all boys vs. all girls)	135 (9.9) vs. 194 (15.2)	0.61 (0.48–0.77; *p* < 0.001)
Female PBTS vs. female controls	24 (11.0) vs. 170 (16.1)	0.64 (0.41–1.02; *p* = 0.058)
Male PBTS vs. male controls	12 (5.1) vs. 123 (10.9)	0.44 (0.24–0.82; *p* = 0.007)
Mothers' education		*p* < 0.001
Fathers' education		*p* < 0.001
Differences between PBTS age groups		*p* = 0.838
High‐grade tumors vs. low‐grade tumors	*N* < 5	—
High‐grade tumors. vs. all controls	*N* < 5	—
Low‐grade tumors vs. all controls	33 (8.9) vs. 293 (13.4)	0.63 (0.43–0.92; *p* = 0.016)
Low‐grade astrocytomas vs. all controls	20 (12.8) vs. 293 (13.4)	0.95 (0.58–1.54; *p* = 0.840)
Optic pathway gliomas vs. all controls	*N* < 5	—
Craniopharyngiomas vs. all controls	*N* < 5	—
Neuronal and mixed neuronal‐glial tumors vs. all controls	*N* < 5	—

*Note*: Number (*N*), percentage, odds ratio (OR), 95% confidence interval (CI), and significance (*p*) for differences between pediatric brain tumor survivors (PBTS) and controls.

### 4.4 Occupational outcomes, year‐by‐year from 2011 to 2016

**FIGURE 1:**
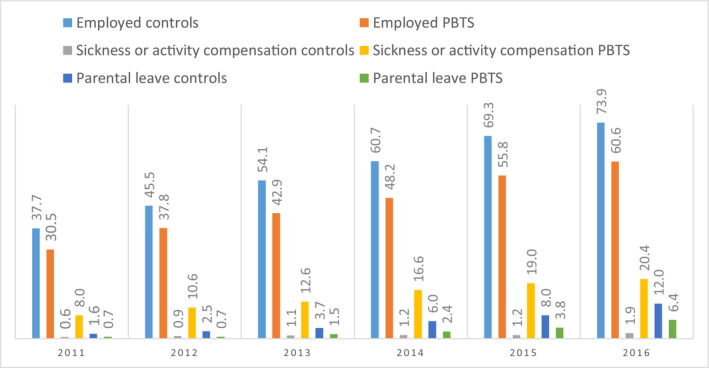
Percentage of controls and pediatric brain tumor survivors (PBTS) who were employed, received sickness or activity compensation, and/or were on parental leave annually between 2011–2016.

In the year 2011, PBTS and controls in the current study were aged between 15–23 years and 20–28 years in 2016. As expected, there was an increase every year for both PBTS and controls for all three parameters (Figure 1). The proportion of PBTS being employed was significantly lower compared with controls for each year (2011 *p* = 0.004; 2012 *p* = 0.003; 2013 *p* < 0.001; 2014 *p* < 0.001; 2015 *p* < 0.001; 2016 *p* < 0.001), and PBTS received sickness or activity compensation at least ten times more often than controls each year (2011 *p* < 0.001; 2012 *p* < 0.001; 2013 *p* < 0.001; 2014 *p* < 0.001; 2015 *p* < 0.001; 2016 *p* < 0.001). Around twice as many controls as PBTS were on parental leave each year, and the difference was significant from 2012 onwards (2011 *p* = 0.143; 2012 *p* = 0.015; 2013 *p* = 0.020; 2014 *p* = 0.002; 2015 *p* = 0.002; 2016 *p* = 0.001).

## 5 DISCUSSION

In this study, we have explored occupational outcomes of teenagers and young adults treated for a brain tumor in childhood. There were significant differences between PBTS and controls for all assessed variables. The largest differences were observed for the variable, sickness or activity compensation, with PBTS being on average more than 11 times more likely to receive such benefits compared with controls. These results are in line with a previous study from Finland^47^ where the OR for early retirement was shown to be 14.80 times higher for those treated for a brain tumor in childhood compared with controls. Early retirement is to some extent comparable to the Swedish sickness or activity compensation. In the Finnish study, no significant differences were observed between females and males, which is in line with our results, but unlike our results, they found significant differences between cases treated at different ages. Our subgroup analyses showed that the highest differences for obtaining sickness or activity compensation were seen for those treated for high‐grade tumors or low‐grade craniopharyngiomas. Both these groups had an OR more than 24 times higher than controls, indicating a high burden of late complications. Although it is remarkable that the morbidity generated by the low‐grade tumor craniopharyngioma and/or its treatment, is in the same range as that of high‐grade tumors, this is also supported by earlier observations.^48^ PBTS treated for optic pathway gliomas had an OR more than 11 times higher compared with controls for receiving sickness or activity compensation. Moreover, PBTS treated for low‐grade astrocytomas and neuronal and mixed neuronal‐glial tumors, the subgroup associated with the lowest level of morbidity as judged by occupational outcomes, still had an OR six times higher than controls for receiving sickness or activity compensation. In summary, there is great variation in outcomes for different subgroups of PBTS, as judged by sickness or activity compensation, but even those least affected were more than six times more likely to have obtained such benefits. It is problematic that so many PBTS, with many years left to live, appear unable to work already at a young age.

Also for employment we found significant differences between PBTS and controls. Only 73.9% of PBTS had been employed at any time during the years 2005–2016, compared to 88.5% in the control group. PBTS treated for high‐grade tumors had the lowest rate of employment, 57.5%, followed by the low‐grade tumor subgroups craniopharyngiomas (64.5%), optic pathway gliomas (70.0%), neuronal and mixed neuronal glial tumors (76.3%) and low‐grade astrocytomas (81.4%). These results are in line with both a previous Swedish study with data from individuals born 1963–1976^49^ and other international studies,^50^, ^51^ showing that PBTS are less likely to be employed than healthy comparators. However, one study^47^ did not find an elevated risk of unemployment. When looking at the employment rates year by year in the current study, the differences between PBTS and controls were stable and increased 2011–2015, followed by a slight decline 2015–2016. However, it is possible that observed differences between PBTS and controls could decrease over time in later years. For example, a Japanese study^50^ found that individuals treated for childhood brain tumors were significantly older by the time of their first employment compared with controls. This is also in line with a German study^52^ of children treated for different cancers.

For parental leave, there were significant differences between PBTS and controls, with fewer PBTS having been on parental leave. These results are in line with previous observations showing that PBTS had a lower rate of cohabitation compared with controls, and were also unmarried to a higher rate as adults.^32^, ^33^ As with employment, it is possible that PBTS will have children later in life, and that the differences would have been smaller if we had performed the analyses later, when the participants were older. For both employment and parental leave, parents' education had a significant impact but it was the same for PBTS and controls.

Unexpectedly, neither age at diagnosis, nor sex, influenced occupational outcomes. In our three previous studies of almost the same cohort, comparing their school grades from school year nine and national tests from the same year,^28^, ^29^, ^43^ we found that children treated at a young age, especially females, were at particular risk of poorer academic performance, but this was not the case for occupational outcomes in the current study. However, the differences between PBTS treated for high‐grade or low‐grade tumors were more evident in this study, when the participants were older and late complications had become more evident over the years. Nevertheless, our results still show that both PBTS treated for high‐grade and low‐grade tumors are at risk for late complications that affect their daily life.

### 5.1 Implications

The main implication of our results is that it is essential to follow up all PBTS, not just those known to be at high risk. Even though there are PBTS with few or less severe late complications, it is important to identify both those with severe complications and to characterize the challenges experienced by those with few or less severe complications, for example fatigue, impaired working memory, and reduced processing speed – early on and provide appropriate support.

The risk of being unemployed in Sweden is particularly high for two groups of young adults, those without education from upper secondary school and those with a disability.^53^ As we have shown in one of our previous studies,^28^ PBTS are at high risk of not being admitted to upper secondary school/high school. Other studies have shown that the ability to work is impaired for many PBTS.^50^, ^54^ Thus, this is a group with a double risk of unemployment that need to be closely monitored even before they reach the age when they enter the labor market, as unemployment may have a negative impact on their life and well‐being.^55^

### 5.2 Strengths and limitations

The major strength of this study is that it is based on data from all children born between 1988 and 1996 included in the Swedish Childhood Cancer Registry, and this registry comprises information about 93.2% of all children in Sweden treated for cancer. We also have solid information about background factors, such as sex, tumor grade, and parents' education.

A limitation of the study is that we have no information about their outcomes in a long‐term perspective, as the data are from recent years. Repeating the study when more time has passed and including supporting variables, such as income and marital status, would shed further light on the development of late complications and occupational outcomes. Another limitation is that the most severely impaired survivors (e.g., those with an IQ below 70) may have been excluded, since the schools for children with disabilities have a different grading system and these grades are not included in the registries. Hence, our results likely underestimate the effects, albeit to a minor extent since the number of missing children is low. Moreover, information about radiation therapy was unfortunately incomplete or missing in our data, and therefore we used diagnosis as a surrogate marker for treatment intensity.

## AUTHOR CONTRIBUTION

**Malin Lönnerblad** Project administration, conceptualization, analysis, interpretation of the data, visualization, writing–original draft, writing–review and editing. **Eva Berglund** Writing–review and editing. **Maria Åberg** Writing–review and editing. **Klas Blomgren** Interpretation of the data, writing–original draft, writing–review and editing.

## Data Availability

The data that support the findings of this study are available from the Swedish Childhood Cancer Registry and Statistics Sweden. Ethical approval for this study was given by the Regional Ethical Review Board in Stockholm (no. 2017/995‐31/5). Restrictions apply to the availability of these data, which were used under license for this study.
